# Profiling the resistome and virulome of *Bacillus* strains used for probiotic-based sanitation: a multicenter WGS analysis

**DOI:** 10.1186/s12864-025-11582-1

**Published:** 2025-04-18

**Authors:** Francesca Bini, Irene Soffritti, Maria D’Accolti, Eleonora Mazziga, Julio Diaz Caballero, Sophia David, Silvia Argimon, David M. Aanensen, Antonella Volta, Matteo Bisi, Sante Mazzacane, Elisabetta Caselli

**Affiliations:** 1 Section of Microbiology, Department of Environmental and Prevention Sciences, and LTTA, University of Ferrara, Ferrara, 44121 Italy; 2 CIAS Research Center, University of Ferrara, Ferrara, 44122 Italy; 3 Centre for Genomic Pathogen Surveillance, Big Data Institute, University of Oxford, Oxford, UK

**Keywords:** Resistome, Virulome, WGS, Probiotics, Sanitation, *Bacillus*

## Abstract

**Background:**

Healthcare-associated infections (HAIs) caused by microbes that acquire antimicrobial resistance (AMR) represent an increasing threat to human health worldwide. The high use of chemical disinfectants aimed at reducing the presence of pathogens in the hospital environment can simultaneously favor the selection of resistant strains, potentially worsening AMR concerns. In the search for sustainable ways to control bioburden without affecting this aspect, probiotic-based sanitation (PBS) using *Bacillus* spp. was proposed to achieve stable reduction of pathogens, AMR, and associated HAIs. Although *Bacillus* probiotics are classified as nonpathogenic, comprehensive data about the potential genetic alterations of these probiotics following prolonged contact with surrounding pathogens are not yet available. This study aimed to assess in depth the genetic content of PBS-*Bacillus* isolates to evaluate any eventual variations that occurred during their usage.

**Results:**

WGS analysis was used for the precise identification of PBS-*Bacillus* species and detailed profiling of their SNPs, resistome, virulome, and mobilome. Analyses were conducted on both the original PBS detergent and 172 environmental isolates from eight hospitals sanitized with PBS over a 30-month period. The two species *B. subtilis* and *B. velezensis* were identified in both the original product and the hospital environment, and SNP analysis revealed the presence of two clusters in each species. No virulence/resistance genes or mobile conjugative plasmids were detected in either the original PBS-*Bacillus* strain or any of the analyzed environmental isolates, confirming their high genetic stability and their low/no tendency to be involved in horizontal gene transfer events.

**Conclusions:**

The data obtained by metagenomic analysis revealed the absence of genetic sequences associated with PBS-*Bacillus* and the lack of alterations in all the environmental isolates analyzed, despite their continuous contact with surrounding pathogens. These results support the safety of the *Bacillus* species analyzed. Further metagenomic studies aimed at profiling the whole genomes of these and other species of *Bacillus*, possibly during longer periods and under stress conditions, would be of interest since they may provide further confirmation of their stability and safety.

## Background

Persistent microbial contamination of the hospital environment has been associated with an increased risk of acquiring healthcare-associated infections (HAIs), which represent an increasing global concern [1, 2]. HAIs affect over 4.3 million people per year in the European Community alone, causing an estimated 91,000 deaths largely attributable to six main HAI types (healthcare-associated pneumonia, urinary tract infection, surgical site infection, *C. difficile* infection, neonatal sepsis and primary bloodstream infection), according to a study based on European Centre for Disease Control (ECDC) data from the 2011–2012 Point Prevalence Survey (PPS) [3, 4]. HAIs are a major public health problem in Italy, according to the ECDC PPS-2024, affecting 9.8% of hospital inpatients, whereas the median prevalence is 6.8% across all EU/EEA countries [3].

The control of hospital bioburden, which is essential for preventing HAI risk, has been addressed through the use of chemical disinfectants, the use of which has further increased during the COVID-19 pandemic in an attempt to prevent SARS-CoV‐2 transmission through contaminated surfaces [5, 6]. While disinfectants are rapidly effective, they have several important limitations, including temporary action [7, 8], high environmental impact, and a tendency to increase the degree of antimicrobial resistance (AMR) in potential human pathogens [9]. The phenomenon of cross-resistance between disinfectants and antimicrobials has been reported for several disinfectants and is associated with a further increase in AMR during the pandemic period [5, 10, 11]. On the other hand, according to ECDC data, HAIs are increasingly caused by difficult-to-treat pathogens, which can become potentially fatal for hospitalized inpatients due to their high levels of AMR [3, 12–15]. Indeed, AMR has become one of the major threats to human health, emerging and spreading, particularly in hospital environments, as a result of the selective pressures exerted by the widespread use of disinfectants and antibiotics in these settings [16]. Consequently, the microorganisms that persistently contaminate the hospital environment are often drugs, multidrug (MDR), or totally drug resistant (TDR), posing a significant threat to public health, as they can cause severe/lethal infections and spread to other sanitary and nonsanitary settings, including community environments and mass transport [17–20].

On the basis of the current spread of AMR, the World Health Organization (WHO) warned about the risk of future AMR pandemics [21] and estimated that at least 10 million deaths per year may occur by 2050 if no urgent concrete global action is taken [22]. Consistent with this, many countries have taken proactive measures to fight AMR, introducing policies to reduce the general use of antibiotics in healthcare and nonhealthcare environments, according to the “One Health” approach [23], supporting surveillance programs, and introducing procedures to prevent the spread of resistant microorganisms [24, 25]. The “One Health” principles are based on the understanding that to maintain human health, both animal and environmental health must be taken into account. Controlling all these factors may be necessary to effectively achieve health-related goals since microorganisms, AMR, and pollutants spread in a circle within these areas. In particular, addressing AMR and infectious risk control through environmentally sustainable approaches may prevent further soil and water pollution, preserve animal and plant health, and ultimately human health itself. These principles have been widely recognized by WHO in the last decade, becoming a key reference towards AMR and infection control [26].

Since the control of microbial contamination in healthcare settings is an essential practice to prevent HAIs and ensure patient safety [3, 27], several recent studies have focused on the search for new potential long-term sanitation approaches, ideally without worsening earth/water pollution and AMR concerns.

To achieve this goal, probiotic-based sanitation (PBS) methods were set up and tested, revealing their ability to stably abate hospital pathogens in treated environments, with no accompanying selection of resistant strains. In particular, the Probiotic Cleaning Hygiene System (PCHS^®^) is a patented system based on the use of a fully eco-labeled detergent containing spores of selected probiotics belonging to the *Bacillus* genus (namely, the species *B. subtilis*, *B. pumilus*, and *B. megaterium*). Upon dilution in water and spread on surfaces during the cleaning procedure, the spores germinate, generating vegetative bacteria, which are able to remove organic dirt by enzymatic digestion while displacing surrounding pathogens via competitive mechanisms [28]. This type of sanitation has been shown to reduce surface pathogens 80% more than chemical disinfectants without selecting resistant strains but rather decreasing the prevalence of preexisting AMR up to 99.9%, resulting in a > 50% decrease in HAI incidence [8, 29–35]. PCHS has also been shown to provide long-lasting decontamination from enveloped viruses in vitro [8], and its usage was tested during the COVID-19 pandemic, showing significant effectiveness against SARS-CoV-2 in both sanitary and non-sanitary environments [20, 36].

Similar results were obtained when other PBS formulations, including *Bacillus* probiotics, were used, revealing their generally greater effectiveness in controlling nosocomial pathogens and AMR [37] than disinfectants or other detergents [38]. On the basis of these data, a recently released recommendation by the Robert Koch Institute Commission for Hospital Hygiene and Infection Prevention included probiotic cleaning as a sustainable way to provide a long-term stable microbiome without favoring the development of cross-resistance to antibiotics [39].

However, owing to the high susceptibility and fragility of hospitalized patients, a major aspect to consider for any hospital sanitizing procedure is the safety of use to ensure that it poses no risk for hospital patients. Owing to the use of live microorganisms, the safety of the use of PBS represents a critical point to assess.

*Bacillus* probiotics are classified as generally regarded as safe (GRAS) by the Food and Drug Administration [40] and by the European Food Safety Authority (EFSA) [41]. According to the recent EFSA document, the requirements to be included in the ‘‘Qualified Presumption of Safety’’ (QPS) group include a lack of pathogenicity and the absence of acquired resistance genes [42]. Most *Bacillus* species, including the *B. subtilis* group, *B. pumilus*, *B. megaterium*, *B. velezensis*, and other *Bacillus* species, meet these requirements for QPS [43]. *Bacillus* spp. have been used safely for decades in many applications aimed at preserving human, animal, and plant health [28, 31], and studies of microbiological surveillance during their use as sanitizers revealed the absence of *Bacillus*-induced infections and *Bacillus*-positive clinical samples in more than 30,000 tests, corresponding to approximately 90,000 hospitalized patients, in the structures generated via PCHS [32]. Additionally, PCR analyses did not reveal any newly acquired R genes in approximately 500 PCHS-*Bacillus* strains derived from the treated hospitals [32, 35].

However, an in-depth characterization of the whole genome content of PCHS-*Bacillus* before and after prolonged contact with pathogens on treated surfaces is still lacking. Instead, investigation on *Bacillus* genetic stability would be essential to exclude potential harmful alterations linked to genetic exchange with close bacteria, especially when *Bacillus* are used for PBS in hospital settings. This due to the presence in this context of several resistant and virulent microbes, which may transfer resistance or virulence genes to *Bacillus* probiotics. Moreover, *Bacillus* genus include several species with different characteristics [44], and there is growing need to evaluate the individual *Bacillus* species and strains on a case by case basis and necessity [45]. On the other side, hospitalized patients are particularly susceptible to infections by every microorganism, thus the assessment of *Bacillus* genetic stability appears even more needed in this setting. In addition, safety studies have been mostly performed for the use of *Bacillus* probiotics as food additives in humans, also recently [46], but they are lacking when they are used as sanitizers in PBS. Addressing this gap in the literature and providing evidence of long-term stability of *Bacillus* included in PBS would support their safe use in hospitals and consequently in many non-sanitary and less complicated environments.

Thus, this study aimed to assess their stability with respect to both resistance and virulence gene acquisition during their use as sanitizers, to characterize their whole resistome and virulome, and to identify their original features and any putative changes that may have occurred following contact with hospital pathogens. For this purpose, we performed whole-genome sequencing (WGS) of PCHS-*Bacillus* strains isolated from eight different Italian hospitals over a 30-month period to verify their genetic stability, thereby validating their safety for use in associated cleaning systems.

## Methods

### Study design

The analysis was performed on a retrospective collection of approximately 200 *Bacillus* isolates collected in eight Italian healthcare settings (HSs), originally enrolled in previous studies aimed to test PCHS effectiveness, from 20 January 2015 to 28 June 2017, for a total period of 30 months [34, 35]. Hospitals were located in different geographical areas of Italy, including the following cities: Ferrara (two hospitals: HS-1, a public University-Hospital, and HS-2, a private hospital), Feltre (HS-3), Foggia (HS-4), Pavia (HS-5), Rome (HS-6), Tolmezzo (HS-7), and Vigevano (HS-8). During the study period, PCHS was used daily in the general medicine wards of all enrolled hospitals as a substitute for conventional chemical sanitation based on chlorine products and alcohol-based disinfectants. The product used in PCHS consisted of an eco-labeled detergent containing anionic and non-ionic detergents with neutral pH and including 5 × 10^7^ spores of probiotics per ml (Copma Scpa, Ferrara, Italy).

As a part of PCHS studies, each enrolled HS was monitored by performing regular microbial environmental sampling by collecting surface samples seven hours after sanitation [7, 34, 35]. Sampling campaigns were performed at 3–24 months after PCHS introduction, with time intervals depending on the type of study, which included two single-center studies (in both Ferrara HSs) and one multicenter study comprising all the other hospitals. Briefly, from 3 to 24 months in HS-1, from 2 to 6 months in HS-2, at 3, 9, and 12 months in HS-3, and at 3 and 6 months in all the remaining HSs. For all the enrolled HSs, samples were collected in duplicate via replicate organism detection and counting (RODAC) agar plates from sinks, floors, and bed footboards of ward rooms [34].

*Bacillus* colonies were isolated on RODAC plates containing Baird‒Parker agar medium, as previously described [20, 34]. The individual colonies of *Bacillus* spp. were then amplified via 24-hour incubation in 5 ml of tryptic soy broth (TSB) at 37 °C under mild agitation. Microbial cells were then collected by centrifugation at 14,000 × g for 5 min and stored at -80 °C until use. Similarly, individual *Bacillus* colonies from the original PCHS detergent were isolated on Baird‒Parker RODAC plates [35]. Molecular analyses were performed on both the environmental PCHS-*Bacillus* isolates and the original PCHS-*Bacillus* strains.

### DNA extraction

Total DNA was extracted from microbial pellets by the QIAmp UCP Pathogen Mini Kit (Qiagen, Hilden, Germany) following the manufacturer’s instructions [33, 34]. The DNA purity of the *Bacillus* isolates was estimated via spectrophotometric readings at wavelengths of 260/280 nm via a NanoDrop spectrophotometer (Nanodrop Technologies Inc., USA). The concentration was further determined by using a Qubit fluorometer (Invitrogen, Carlsbad, CA, USA). Prior to subsequent analyses, the amplifiability of each extracted DNA sample was checked via a panbacterial PCR designed in the conserved region of the 16 S rRNA gene, as described previously [35].

### Identification of *Bacillus* species

Each *Bacillus* isolate, either from PCHS detergent or from the hospital environment, was initially identified via PCR and amplicon sequencing, as previously described [35]. Briefly, 10 ng of extracted DNA was amplified via PCR of the 16 S rRNA coding gene, and the resulting 400 bp amplicons were sequenced and compared with the BLAST Bacillus sequences database (https://blast.ncbi.nlm.nih.gov/Blast.cgi) [35]. On the basis of these results, only the environmental isolates recognized as *Bacillus* species derived from the PCHS detergent were included in the subsequent analyses by WGS.

### Whole genome sequencing (WGS) analysis

Each *Bacillus* isolate from both the PCHS detergent and the hospital environment was subsequently analyzed via whole-genome sequencing (WGS) at the Oxford Genomics Centre (University of Oxford, UK). All collected samples were processed simultaneously. Briefly, 1000 ng of DNA was used for sequencing analysis, library construction was performed via NEBNext Ultra reagent kits (NEB, Ipswich, MA, USA) following a custom automation protocol with a Biomek FX (Beckman Coulter, Indianapolis, IN, USA), and fragmentation of the DNA was performed with an Episonic sonicator (Epigentek, Farmingdale, NY, USA). The sequencing was performed on an SP flow cell lane, an Illumina NovaSeq 6000 (Illumina, San Diego, CA, USA). The sequence reads were output in fastq format.

### WGS quality control and bioinformatic analysis

**Fig. 1:**
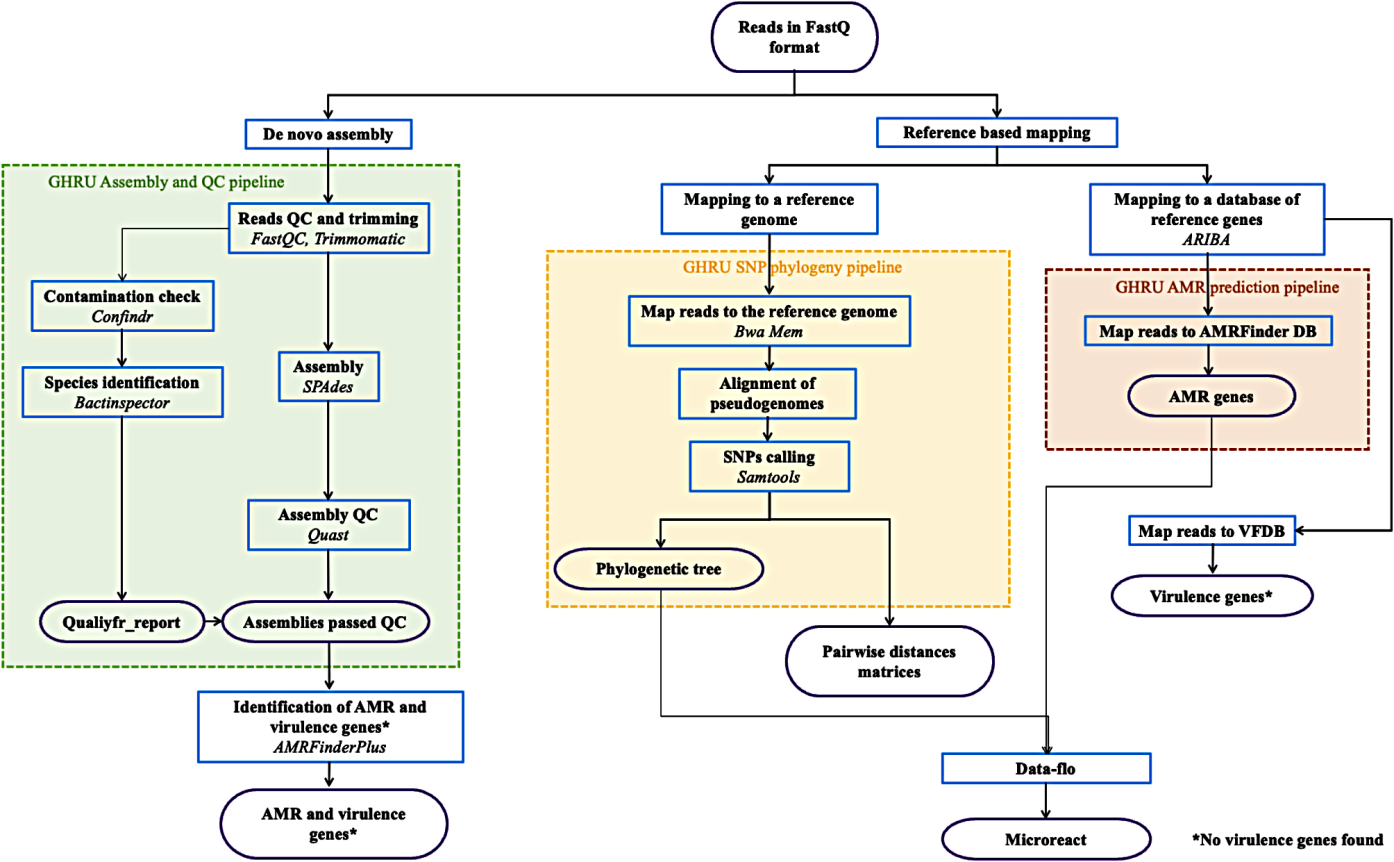
Overview of the bioinformatics workflow used to analyze *Bacillus* genomes. Purple circles represent inputs/outputs. Blue boxes represent tasks that provide a certain functionality (indicated in bold). Dashed boxes represent pipelines. Major software packages used are indicated in italics

All the genome sequence data were processed via specialized pipelines, including de novo assembly, mapping-based SNP phylogenetic methods, and the detection of AMR and virulence determinants, as further explained. Each pipeline used in this study was executed and run at the Biomedical Research Cluster at the University of Oxford and was implemented in Nextflow v21.10.6 and monitored via the Nf-tower interface [47]. The workflow structure is summarized in Fig. 1.

Genomes were first assembled to obtain information on their quality via the Assembly and Quality Control (QC) pipeline [48] developed by the Global Health Research Unit (GHRU) as a part of Genomic Surveillance of Antimicrobial Resistance [49]. The input of the Assembly and QC pipeline was a directory containing matching pairs of fastq files, which were obtained as outputs from the sequencer. The size of the genome was estimated via Mash v2.1 [50], and subsequently, the reads were trimmed to remove the adapters via Trimmomatic v0.39 [51]. The Assembly and QC pipeline used FastQC v0.11.9 to summarize quality metrics before and after adapter trimming and quality control of the reads [52]. The quality-controlled reads were subsequently used to identify the species with Bactinspector v0.1.3 [53], and potential contamination was evaluated via Confindr v0.7.2 [54]. The resulting quality-controlled reads were used with SPAdes v3.15.3 for the assembly [55], and then Quast v5.0.2 was used to summarize the assembly metrics of each genome [56]. Finally, all the quality metrics were combined via MultiQC v1.11 and Qualifyr v1.4.6 to generate a report with the results of all the assembly pipeline steps for all the genomes [57, 58]. The *Bacillus* genome assemblies were subsequently uploaded to and analyzed via PathogenWatch [59] to assess the species ID and MLST (multilocus sequence typing) and to select the most appropriate reference strain for generating subsequent reference-based alignments. To this end, the SNP phylogenetic pipeline [60] was used to map sequence reads to selected reference genomes via BWA-MEM v0.7.17 [61]. Specifically, two reference genomes were used for PCHS-*Bacillus* species identification: *B. subtilis subsp. inaquosorum* strain DE111 [GenBank accession number: CP013984.1] [62] and *B. velezensis* strain BT2.4 [NCBI RefSeq accession number: NZ_CP085505.1] [63]. SAMtools v1.9 and SNP sites v2.4.1 were used to identify SNPs [64, 65], which were subsequently used to generate maximum likelihood phylogenetic trees with 1000 bootstrap via IQ-Tree v1.6.8 [66].

During the PathogenWatch analysis, plasmid replicon sequences were also identified via Inctyper, a tool implemented in PathogenWatch that uses PlasmidFinder v2.0.1 to identify contigs containing plasmid Inc. genes [67, 68].

The prediction of AMR and virulence determinants was performed using both the AMRFinderPlus v3.10.45 tool, which uses a reference database that can use assemblies as input for detection, and the ARIBA v2.14.4 tool, which instead allows sequence reads to be mapped onto several reference gene databases [69, 70]. The AMRFinderPlus v3.10.45 tool using the AMRFinder database version 2022-10-11.2 identified AMR genes and additional gene classes, including virulence factors, whereas the ARIBA v2.14.4 tool was implemented via the AMR prediction pipeline using the AMRFinder database version 2022-01-31 and the PointFinder database version 2021-02-17 in parallel to determine the presence of AMR genes [71]. Additionally, the ARIBA v2.14.4 tool was used with VFDB version 2020-01-06 for the prediction of virulence factors [72].

Finally, pairwise SNP distances were calculated for all *Bacillus* isolates by using the Pairsnp v.0.0.7 tool [73] to identify putative changes in the *Bacillus* genomes.

The statistical analysis of the data obtained for both phylogenetic and resistance/virulence analyses was carried out using quality metrics implemented in each of the software packages used, similar to what previously reported [74, 75].

### Data visualization

To obtain actionable information, epidemiologic and genomic data needed to be integrated into the final visualization. For this purpose, we used Data-flo [76] to combine the genomic *Bacillus* data and metadata, including geographic data, with the information obtained from the AMR gene analysis. The merged data were uploaded into the visualization tool Microreact [77] along with the phylogenetic trees for *B. subtilis* and *B. velezensis*.

## Results

### *Bacillus* isolates

Approximately 200 *Bacillus* isolates previously collected over a 30-month period from eight Italian hospitals routinely sanitized by PCHS [7, 34, 35, 78] were included in the molecular analysis. In addition, individual *Bacillus* colonies directly collected from the original PCHS detergent were also analyzed. Initial identification of each *Bacillus* isolate was performed via PCR and amplicon sequencing to establish their putative species. Two species were identified in the original detergent, *B. subtilis* and *B. pumilus*, although they were expected to also contain the species *B. megaterium* on the basis of the composition label of the product. Similarly, those two species, *B. subtilis* and *B. pumilus*, were also recognized in all the environmental isolates, as judged by the PCR sequencing results.

Subsequent de novo sequencing further revealed that 172/200 isolates were indeed derived from the PCHS detergent and were thus enlisted for subsequent WGS analysis, together with the two original PCHS-*Bacillus* strains. Overall, 69 isolates were derived from HS-1, 19 from HS-2, 36 from HS-3, 11 from HS-4, 9 from HS-5, 10 from HS-6, 10 from HS-7, and 8 from HS-8. Table 1 summarizes the number of sampling campaigns and the total number of *Bacillus* isolates from each of the enrolled HSs, as well as the surface type from which they were collected.

**Table 1:** Breakdown of the 172 *Bacillus* selected samples by hospital, City, sampling campaigns, months after PCHS application, and hospital surfaces

Healthcare setting (HS)	*N*. of samplings	Sampling time^1^	*N*. of Bacillus isolates			
			Sink	Floor	Bed footboard	Total
HS-1	8	3,6,9,12,15,18,21,24	24	22	23	69
HS-2	4	2,4,5,6	6	7	6	19
HS-3	3	3,9,12	11	14	11	36
HS-4	2	3,6	3	4	4	11
HS-5	2	3,6	3	3	3	9
HS-6	2	3,6	2	5	3	10
HS-7	2	3,6	3	4	3	10
HS-8	2	3,6	2	4	2	8

^1^ Sampling time is expressed as month number after the first PCHS application

### Genome assembly and quality control

Among the 172 genomes derived from PCHS-*Bacillus* isolates, 23 (23/172, 13%) did not pass the assembly quality control (QC) because of the presence of multiple *Bacillus* strains or contamination by genetic material from other species, including *Enterobacter hormaechei* and *Bacillus licheniformis*. Consequently, only the 149 genomes that passed QC, together with the two genomes derived from the detergent, were included in the downstream analyses.

First, species assignment was performed via Bactinspector software, which is based on sequence assembly. The results revealed that the collected isolates included two species of *Bacillus* spp., *B. subtilis* (105/149, 70.5%) and *B. velezensis* (44/149, 29.5%). These data were confirmed via the web application Pathogenwatch, which allowed further distinguishing the *B. subtilis* genomes into two different sequence types (ST), ST13 (101/105, 96.2%) and ST47 (4/105, 3.8%). Additionally, the strains derived directly from the PCHS detergent, analyzed in parallel, were identified as *B. subtilis* and *B. velezensis*, indicating that the previous species identification of *B. pumilus* via PCR amplicon sequencing was not precise.

### Genetic diversity among *Bacillus* isolates

**Fig. 2:**
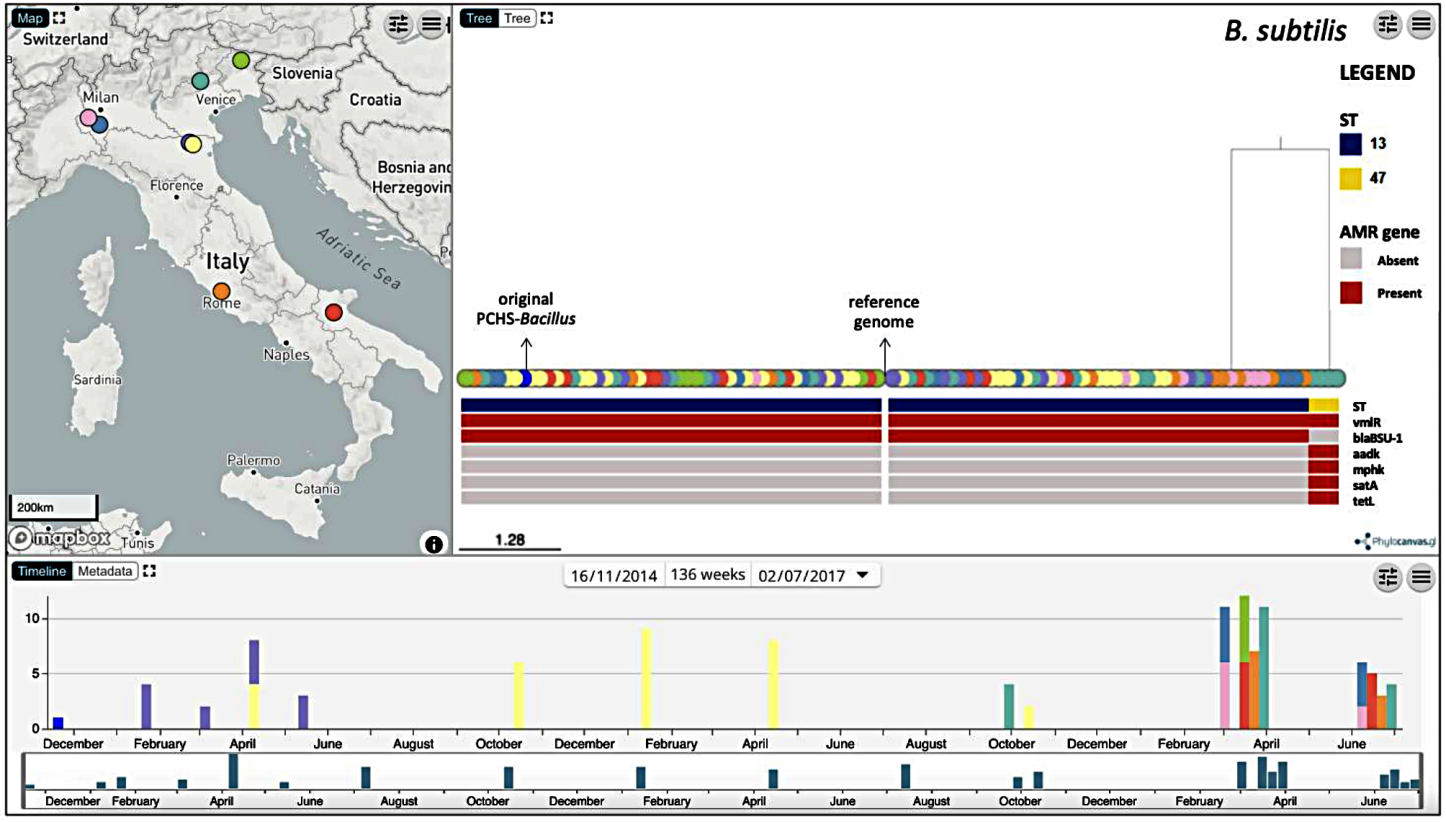
Microreact visualization of *B. subtilis* genomes linked to their geographical and temporal data. The phylogenetic tree was inferred from core-genome SNPs obtained by mapping each genome to reference genome *B. subtilis* DE111. The tree nodes are colored depending on the hospital (map) and annotated with the distribution of ST and AMR genes. The arrows indicate the reference genome and the original PCHS-*Bacillus* strain directly isolated from the detergent

**Fig. 3:**
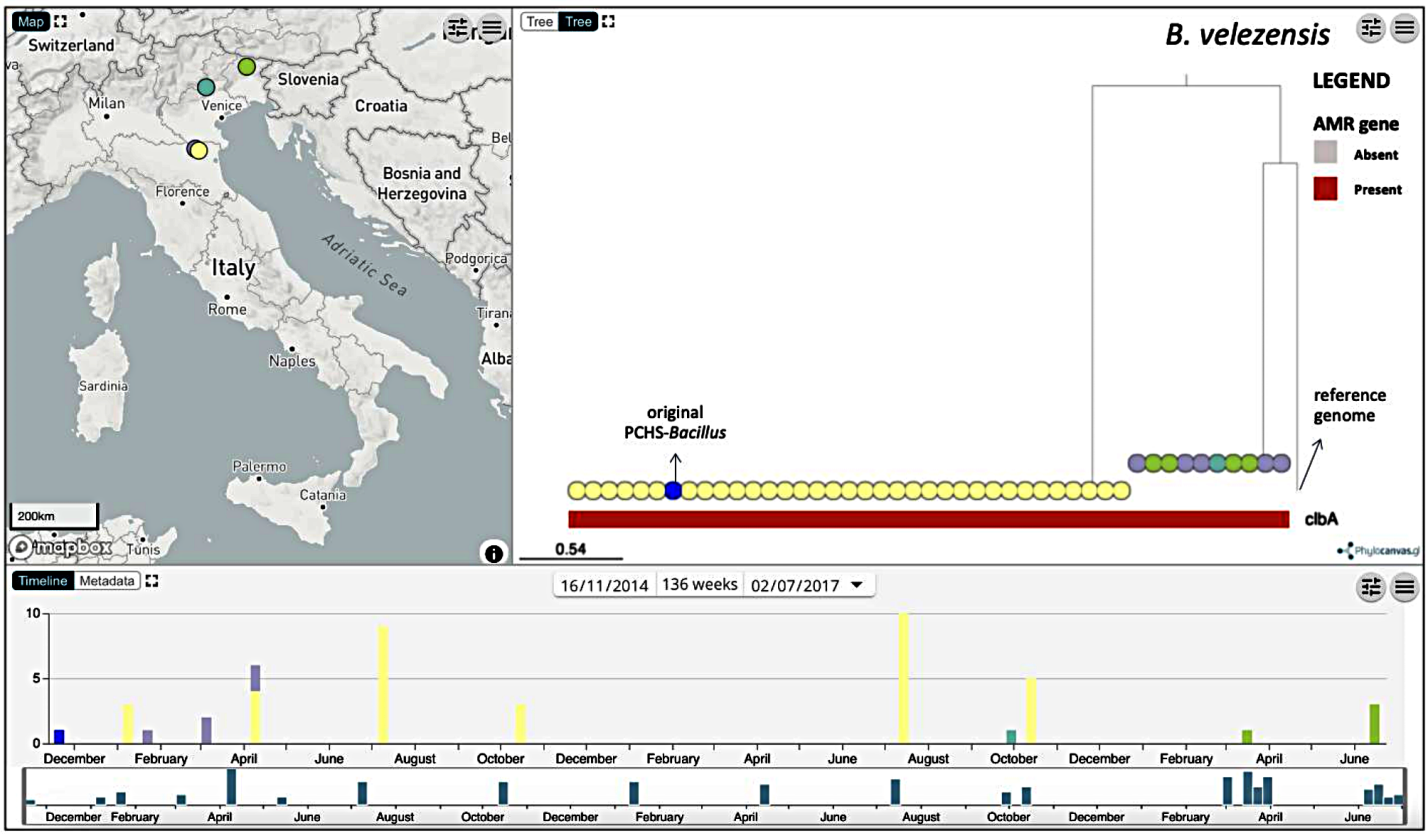
Microreact visualization of *B. velezensis* genomes linked to their geographical and temporal data. The phylogenetic tree was inferred from core-genome SNPs obtained by mapping each genome to reference genome *B. velezensis* BT2.4. The tree nodes are colored depending on the hospital (map) and annotated with the distribution of AMR genes. The arrows indicate the reference genome and the original PCHS-*Bacillus* strain directly isolated from the detergent

To investigate the presence of eventual genetic variations among the genomes from the same species, we produced two reference-based alignments comprising the *B. subtilis* and *B. velezensis* genomes only, including the *B. subtilis* and *B. velezensis* strains obtained from the detergent, and inferred two phylogenetic trees (Figs. 2 and 3). The phylogenetic analysis confirmed the presence of two different genotypes identified with MLST for *B. subtilis*, clustering the genomes into the two sequence type groups ST13 (101/105 genomes) and ST47 (4/105 genomes). Among them, the major ST13 group included samples collected from all surfaces of eight HSs at multiple different time points in the 30-month period, whereas the ST47 group was derived entirely from HS-3 (floors and bed footboards) between March and June 2017. The *B. subtilis* genome derived directly from the detergent belonged to the ST13 group (102 total samples).

Similarly, despite the lack of an MLST scheme, the phylogenetic tree also revealed two distinct groups among the *B. velezensis* genomes, a major group (35/45, 77.8%) and a minor group (10/45, 22.2%), as shown in Fig. 3. The major clade of *B. velezensis* (34/44 genomes) was derived from HS-1 in a period of 22 months, whereas the minor clade (10/44 genomes) was derived from samples collected from HS-7 (4 samples) between March and June 2017, from HS-3 (1 sample) in September 2016, and from HS-2 (5 samples) between January and March 2015. The *B. velezensis* genome derived directly from the detergent belonged to the major group (35 total samples).

### Detection of SNP differences

Since *Bacillus* genomes are approximately 5 Mbp in length and are characterized by > 90% homology among different species, to better understand the genomic diversity of each group, we quantified the pairwise SNP differences in the reference-based alignments. The 102 (101 environmental isolates and 1 detergent strain) *B. subtilis* ST13 genomes differed by a mean of 1 SNP (range 0–2) (Table 2), whereas the four *B. subtilis* ST47 genomes were identical (0 SNPs). As expected, the SNP distances between genomes from these two STs reflected the high phylogenetic distance (mean of 227,295 SNPs; range 227,158–227,486). The same was observed for *B. velezensis*, where the 10 genomes belonging to the minor cluster differed from those of the major cluster (34 environmental isolates and 1 detergent strain) by a mean of 35,950 SNPs (range 35,822–36,019). Instead, the genomes within each major cluster did not present any SNP differences (0 SNPs).

**Table 2:** Summary of SNP sites found within *B. subtilis* ST13 genomes, as indicated by their position in reference genome DE111 (Accession CP013984.1)

Position^1^	Mutation^2^	*N*. of Genomes^3^	Locus tag /intergenic	Gene product	Codon mutation	Syn/non-syn	Aa change
6385	C→T	1	AN935_00110	Protein xpaC	CGA→GTA	NS	Ala→Val
279,482	G→A	2	AN935_01515	MFS transporter	GCG→GCA	S	Ala
660,533	C→T	3	AN935_03395	ATP-dependent DNA helicase PcrA	ACG→ATG	NS	Thr→Met
1,104,980	A→G	17	AN935_05450	Sodium solute symporter	TGT→TGC	S	Cys
1,975,881	T→C	3	Intergenic	NA	NA	NA	NA

^1^ Reference genome DE111 CP013984.1

^2^ Majority allele→minority allele

^3^ Number of genomes with minority allele

Focusing on the SNP differences between the two original strains and the other genomes of the respective clusters, we found that the original *B. subtilis* ST13 strain (derived directly from the detergent) differed from the 101 *B. subtilis* ST13 isolates by a mean of 1 SNP (range 0–2), whereas the original *B. velezensis* strain showed no SNP difference compared with the 34 *B. velezensis* isolates (0 SNPs).

The minor genetic variability detected within the *B. subtilis* ST13 genomes was further investigated by identifying the loci containing the evidenced variable positions. Five variable positions were identified, four of which occurred within coding regions and one within an intergenic region, detected in a total of 23 genomes out of the 102 analyzed, as three genomes appeared mutated in both the *AN935_05450* and *AN935_03395* genes (Table 2).

**Fig. 4:**
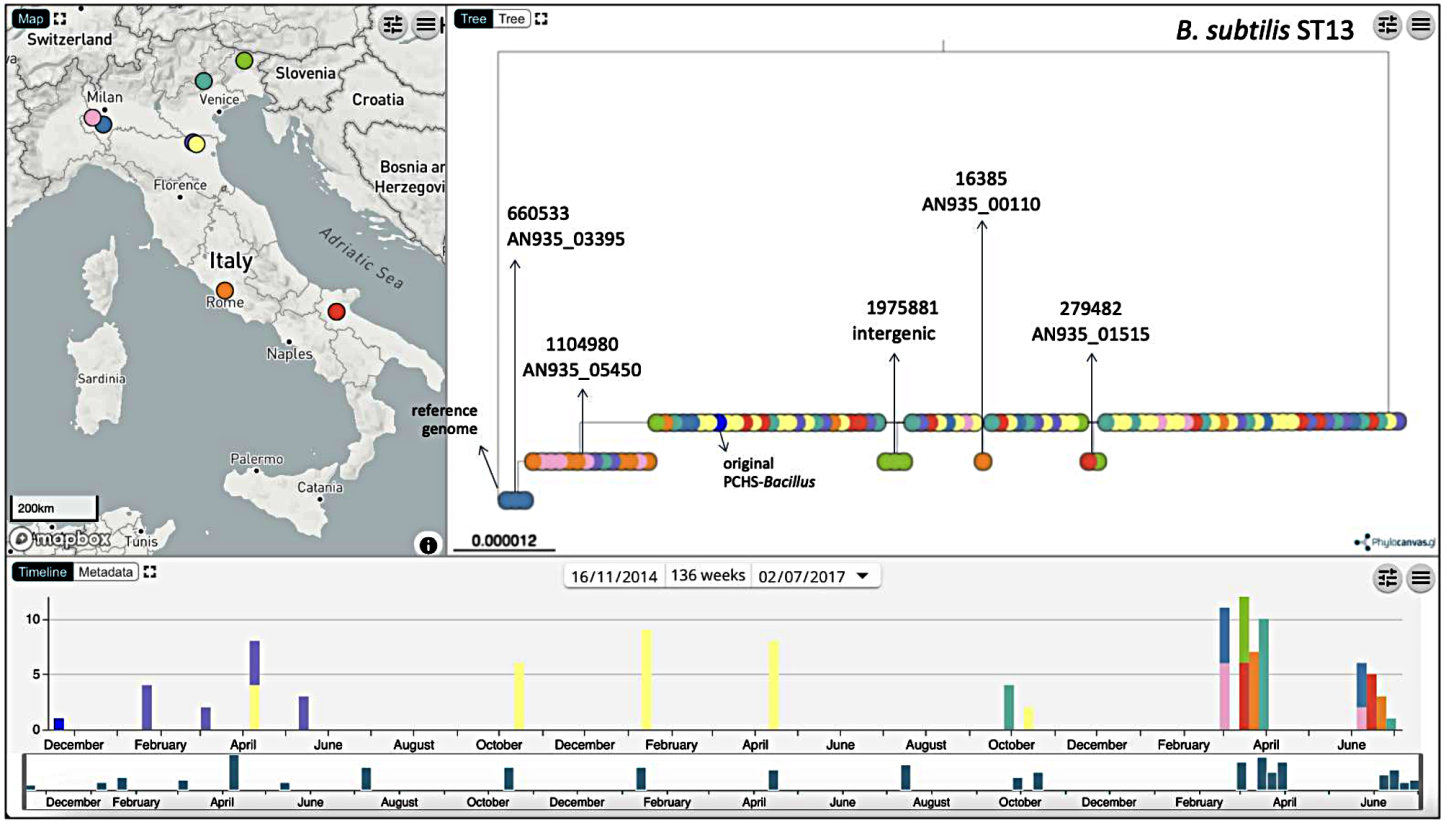
Microreact visualization of *B. subtilis* ST13 genomes linked to their geographical and temporal data. The tree nodes are colored depending on the hospital (map) and annotated with SNPs shared by the genomes indicated by arrows. The arrows also indicate the reference genome and the original PCHS-*Bacillus* strain directly isolated from the detergent

Combining the SNP information obtained from the 102 (101 isolates and 1 original strain) *B. subtilis* ST13 genomes with their geographic data, the results showed that four genomes with mutations in the *AN935_00110* gene were collected from HS-6 and HS-7 hospitals, two genomes with mutations in the *AN935_01515* gene were collected from HS-7 and HS-4, and 17 genomes with mutations in the *AN935_0545*0 gene were collected from five different HSs. Among the 17 genomes with mutations in the *AN935_0545*0 gene, three genomes from HS-5 also had mutations in the *AN935_03395* gene. All samples were collected from different types of surfaces, including floors, sinks, and bed footboards (Fig. 4).

### Detection of antimicrobial resistance genes and virulence factors

The identification of AMR genes via AMRFinderPlus v3.10.45 and the ARIBA v2.14.4 tools produced concordant results, showing that all the *B. subtilis* ST13 isolates, including the *B. subtilis* ST13 original strain, possessed two resistance genes, *blaBSU-1* and *vmlR*, which encode class D beta-lactamases conferring resistance to beta-lactams [79], and an ATPase ribosomal protection protein conferring resistance to streptogramin A and lincosamide antibiotics [80]. The four *B. subtilis* ST47 genomes also harbored *vmlR* but lacked *blaBSU-1*, which instead carried the *aadK* gene, conferring resistance to aminoglycosides [81], and the *tetL* gene, which confers tetracycline resistance [82]. All the detected AMR genes were found to be intrinsic to *B. subtilis* strains (Figs. 2 and 3).

All the analyzed *B. velezensis* genomes, including those of the original *B. velezensis* strain, uniquely contained the *clbA* gene, which confers resistance to some classes of antibiotics, including streptogramins, chloramphenicol, and clindamycin, through the methylation of 23 S rRNA [83]. No other ARGs were detected in any of the 45 *B. velezensis* genomes.

AMRFinderPlus v3.10.45 and the ARIBA v2.14.4 tools produced different results regarding two further resistance genes, *mphK*, encoding resistance to macrolides through the expression of a macrolide phosphotransferase [84], and *satA*, encoding a streptothricin acetyltransferase conferring resistance to streptothricin antibiotics [85]. Specifically, all the analyzed *B. subtilis* genomes, including the *B. subtilis* ST13 original strain, were shown to harbor *mphK* according to the AMRFinderPlus v3.10.45 tool, but closer inspection of the results revealed that the match identity was lower (80.72%) in the ST13 (102 genomes) and higher (97.71%) in the four genomes belonging to ST47. On the other hand, the ARIBA v2.14.4 tool detected a complete *mphK* gene in only the four ST47 genomes, with 97.72% matching identity.

For the *satA* gene, both AMRFinderPlus v3.10.45 and ARIBA v2.14.4 tools detected this gene in the four ST47 genomes but with different matching identities (90.17% and 94.44% with the two tools, respectively), whereas in the ST13 genomes, the AMRFinderPlus v3.10.45 tool did not report any matches, and the ARIBA v2.14.4 tool detected only the absent, partial, or incomplete *satA* gene.

The same tools used with the VFDB database, AMRFinderPlus v3.10.45 tool and ARIBA v2.14.4, allowed the analysis of the whole virulome of all the collected *Bacillus* genomes. The results revealed no virulence genes with either tool, confirming the absence of potentially harmful functions in the examined *Bacillus strains*.

Similarly, the presence of eventual plasmid replicon sequences, potentially carrying AMR or virulence genes, was assessed via Inctyper implemented in PathogenWatch, and no mobile plasmid sequences were detected in any of the analyzed genomes.

## Discussion

The control of microbial bioburden in the hospital environment is crucial to prevent the risk of acquiring HAIs, which represents a global concern [3, 86]. Chemical disinfectants have been used for decades to achieve such control, but despite their immediate effectiveness, they have been reportedly recognized to allow rapid recontamination of sanitized surfaces, finally leading to the persistence of bioburden in the hospital environment [8]. In addition, they have shown high environmental impact and the capacity to favor the selection of disinfectant- and drug-tolerant/resistant strains [5]. Consistent with this, during the COVID-19 pandemic, when chemical disinfection was significantly increased in both sanitary and non-sanitary settings, a significant increase in AMR was recorded [5, 10, 11, 21].

In the search for sustainable systems that are able to provide effective bioburden and HAI control without impacting AMR and pollution, systems based on the use of probiotics as sanitizers (probiotic-based sanitation, PBS) were recently proposed on the basis of studies showing the ability of PBS to provide stable decreases in pathogens and AMR, accompanied in most cases by a significant reduction in associated HAIs [29, 33, 34, 37, 38, 87–92]. The use of probiotics in the hospital environment relies on the fact that they are considered safe for humans [41, 43], and some specific studies have been performed on one of these systems (Probiotic Cleaning Hygiene System, PCHS^®^), reporting no adverse effects or HAIs sustained by PCHS-derived *Bacillus* in hospitalized patients [32] and the good genetic stability of PCHS-*Bacillus*, as confirmed by the absence of newly acquired ARGs over time, as judged by PCR microarray [34, 35].

However, no studies have been performed to assess in detail the eventual genetic alterations of *Bacillus* probiotics following prolonged contact with surrounding hospital pathogens. Since they are living microbes that meet highly susceptible subjects, we wanted to investigate this aspect by using a metagenomic approach able to characterize their whole resistome, virulome, and mobilome before and after application on hospital surfaces.

To this end, two hundred *Bacillus* isolates collected from eight Italian HSs in previous studies on PCHS^®^ application between January 2015 and June 2017 were analyzed via WGS. During that study period, PCHS was applied daily in the general medicine wards of all enrolled HSs as a substitute for conventional chemical disinfection. Environmental samples were collected 3 to 24 months after PCHS application, and PCHS-*Bacillus* individual colonies were isolated after RODAC sampling [34, 35]. Individual colonies were first identified via PCR and amplicon sequencing [35], enabling the identification of 172 *Bacillus* colonies derived from PCHS. Each PCHS-*Bacillus* isolate was then analyzed via WGS for species confirmation and full genetic profiling. Two strains isolated directly from the original PCHS detergent were also included in the analysis.

Among the 172 isolates, 149 passed the assembly QC, together with two genomes derived from the original PCHS*-Bacillus* mix. Species assignment through de novo sequencing revealed the presence of two species, both at the environmental level and in the original detergent, the prevalent *B. subtilis* (70.5%) and the minor *B. velezensis* (29.5%). Notably, although a third species, *B. megaterium*, was also detected on the basis of the composition label of the product, this species was not found in the original detergent or in the treated hospital areas. This might be due to the difficulty of isolating a species that is likely present in a very low percentage proportion compared with the other two species. However, further studies are needed to understand whether *B. megaterium* was indeed contained in the product at the time of the study. Importantly, the isolates initially classified by PCR as *B. pumilus* [35] were instead identified as *B. velezensis*, owing to the deeper sequencing characterization obtained by de novo sequencing. Since *B. pumilus* and *B. velezensis* show a very high degree of homology, as reported in studies regarding *Bacillus* phylogenetic tree reorganization [93], these results highlight the importance of using deep sequencing procedures to obtain precise taxonomic allocation of *Bacillus* species.

By using such a procedure, it was also possible to identify genetic differences among the isolates of the two *Bacillus* species. Specifically, *B. subtilis* genotypes were distinguishable in two different clusters: ST13, representing most isolates (101/105, 96.2%), and ST47, representing a small proportion (4/105, 3.8%). Similarly, the *B. velezensis* isolates could be separated into major (34/44, 77.3%) and minor groups (10/44, 22.7%). The presence of different substrains of PCHS-*Bacillus* isolates, though not expected, could be attributable to their simultaneous presence in the original mixture, which would be compatible with the high degree of homology of substrains in the two selected *Bacillus* species. Though this hypothesis was not explicitly tested, the original strains derived directly from the detergent supported it, as they were shown to belong both to the most represented group of the two species, *B. subtilis* ST13 and *B. velezensis* (major group).

SNP analysis revealed no SNP differences in the genomes belonging to the minor cluster of *B. subtilis* ST47 (0 SNP) or to either cluster of *B. velezensis* (0 SNP). Instead, the 102 genomes classified as *B. subtilis* ST13 (including the original PCHS strain) differed from each other by a mean of 1 SNP (range 0–2). By identifying the SNP-containing loci, five variable positions were found in a total of 23 *B. subtilis* ST13 genomes isolated from different HSs, suggesting that they were probably present in the original mixture rather than occurring within the hospitals themselves. One mutation was detected in an intergenic region, whereas the others occurred in coding regions. Among them, the mutations in the *AN935_00110* gene and the *AN935_03395* gene are non-synonymous substitutions, which can lead to an alteration of the structure of the produced protein, affecting its function. Since the *AN935_00110* gene encodes the xpaC protein, a protein with hydrolase activity, this mutation could affect different activities, including the sporulation process [94]. The *AN935_03395* gene encodes the ATP-dependent DNA helicase PcrA, a specific bacterial helicase that belongs to the UvrD/Rep helicase family with double activity, ssDNA translocation and duplex destabilization; its mutation may thus lead to a loss of protein function, with an inability to hydrolyze ATP and thus to make conformational changes essential for binding to ssDNA [95]. Experimental validation would allow to strengthen these hypotheses.

Considering the period of sample collection and the natural mutation rates of *Bacillus* [96, 97], the absence of substantial genetic variability among the genomes belonging to the same cluster was expected. The results also suggested that the evidenced clusters represented two different sub-strains belonging to the same bacterial species rather than progressive mutations occurring within each species over time.

The search for ARGs in the analyzed genomes, performed with two different tools (AMRFinderPlus and ARIBA), revealed the presence of the genes *blaBSU-1*, *vmlR*, *aadK*, and *tetL* in the *B. subtilis* genomes, revealing that this species can be resistant to beta-lactams, streptogramin A/lincosamide, aminoglycosides and tetracycline [79, 80, 83, 84]. In contrast, all the *B. velezensis* genomes were found to contain a unique intrinsic ARG, the *clbA* gene, which confers resistance to streptogramins chloramphenicol, and clindamycin [83]. Two more genes were indeed detected with different efficiencies by the AMRFinderPlus v3.10.45 and the ARIBA v2.14.4 tools in the analyzed genomes: *mphK*, encoding resistance to macrolides [84], and *satA*, conferring resistance to streptothricin [85]. The *mphK* gene was detected in all *B. subtilis* genomes, including the original PCHS-*Bacillus subtilis* isolate, whereas only ARIBA detected the *mphK* and *satA* genes in the ST47 group. The difference could be due to the different database versions of the two tools used, since the AMRFinderPlus v3.10.45 tool was run with database versions 2022-10-11.2, whereas the ARIBA v2.14.4 tool was run with versions 2022-01-31. This, together with the different inputs used for the analysis, could explain the differences observed for the *mphK* and *satA* genes. Notably, all the detected ARGs were intrinsic to *Bacillus* [98, 99], located on the bacterial chromosome and not on mobile plasmids, suggesting that they could not be easily transferred to other bacteria by HGT. Even more importantly, the results revealed the presence of the same ARGs detected in the original strains of all the *Bacillus* isolates, supporting the conclusion that the PCHS-*Bacillus* strains isolated from treated environments had not acquired any new ARGs from other surrounding bacteria present on hospital surfaces.

Similarly, both the original PCHS-*Bacillus* strains and *Bacillus* isolates were analyzed for the presence of virulence genes. The VFDB database used includes several virulence factors that can be found in both *B. subtilis* and *B. velezensis* species, such as *capBCA* (encoding membrane-associated enzymes facilitating systemic invasion) and hemolysin A (*hlyA*, a pore-forming toxin that possesses hemolytic, cytotoxic, dermonecrotic, and vascular permeability activities) [100, 101]. The results revealed a lack of those and other virulence genes in any of the analyzed genomes, thus confirming the absence of pathogenic potential of the PCHS-*Bacillus* species per se, as well as the absence of newly acquired virulence genes from the surrounding pathogens.

Finally, since the presence of mobile plasmids is associated with AMR and has the general ability to mobilize and exchange genetic material between bacterial cells [68, 102], both original PCHS-*Bacillus* and PCHS-*Bacillus* isolates were analyzed for this aspect. Notably, plasmids have been reported in other *B. subtilis* strains [103], highlighting the differences existing between *Bacillus* strains even within a single species, and the consequent need to carefully check each strain intended for sanitation purposes since the results regarding some strains are not generalizable to the whole species. The results obtained by PlasmidFinder did not evidence any plasmid sequences in any of the analyzed *Bacillus* sequences, confirming that HGT may be unlikely in PCHS-*Bacillus* strains. Indeed, *Bacillus* HGT can also occur via other mechanisms, including transformation-mediated processes [96, 104, 105]. However, the absence of any new acquired resistance/virulence genes in the totality of PCHS-*Bacillus* isolates, unless their continuous contact with pathogens, supports the conclusion that HGT events are unlikely on colonized surfaces, although interspecies HGT has been reported in natural environments [106].

One of the limitations of our study is related to the sample size, which could be enlarged and include samples from different sanitary and non-sanitary settings, from different countries, to provide more sound evidence. In addition, the study’s duration of approximately two years may not be sufficient to exclude possible changes in *Bacillus* on a long-term basis. Similar studies conducted in sanitary environments would provide valuable context, which is lacking. Thus, performing analyses over a longer period may be beneficial for confirming long-term safety. As well, regular monitoring of *Bacillus* genetic content (particularly when used in high-risk sanitary environments) would be important to exclude any possible variations that may occur following contact with MDR pathogens. Another major limitation of our study concerns the robustness of the phylogenetic conclusions, which would be supported by outgroup analysis, which is lacking in our analyses. Longitudinal studies, including different settings, environmental conditions, and application in non-hospital areas, are also needed to deepen the knowledge and pave the way for the safe and effective use of *Bacillus* in many fields.

In addition, it may be of interest to repeat the analyses by using different bioinformatics tools and a full pan-genome tool to check for the presence of any eventual genes not coding for resistance or virulence factors to rule out the acquisition of any type of sequence.

## Conclusions

In conclusion, for the first time, deep WGS analysis provided a complete characterization of the *Bacillus* species used for sanitation purposes in one PBS system (PCHS^®^), supporting their high genetic stability and absence of genes of concern in both the original PCHS-*Bacillus* genomes and those obtained from the surface after prolonged contact with surrounding hospital pathogens. Owing to the lack of newly acquired ARGs, virulence genes, or plasmid sequences detected in any PCHS-*Bacillus* isolate, these data support the safety of their use in the healthcare setting, further highlighting their potential usage in every community environment or even in farms to decrease infectious risk and AMR spread from a “One Health” perspective. Further extensive studies on other strains of *Bacillus* used for similar purposes and claims would be desirable.

## Data Availability

The datasets generated and/or analyzed during the current study are available and visualizable at the URL https://microreact.org/project/bacillusseqproject.
